# Molecular characterization of the malaria vector *Anopheles barbirostris* van der Wulp in Sri Lanka

**DOI:** 10.1186/1756-3305-7-348

**Published:** 2014-07-29

**Authors:** Kanapathy Gajapathy, Pavilupillai J Jude, Sara L Goodacre, Lalanthika BS Peiris, Ranjan Ramasamy, Sinnathamby N Surendran

**Affiliations:** Department of Zoology, Faculty of Science, University of Jaffna, Jaffna, 40000 Sri Lanka; School of Life Sciences, University of Nottingham, Nottingham, NG7 2RD UK; Regional Office, Anti Malaria Campaign, Hambantota, 82000 Sri Lanka

**Keywords:** *Anopheles barbirostris*, Malaria vector, Species complex, Sri Lanka

## Abstract

**Background:**

*Anopheles barbirostris* is a vector of malaria in Sri Lanka. The taxon exists as a species complex in the Southeast Asian region. Previous studies using molecular markers suggest that there are more than 4 distinct clades within the *An. barbirostris* complex in Southeast Asia. The present study characterizes Sri Lankan *An. barbirostris* using mtDNA cytochrome oxidase subunit I (COI) and ribosomal RNA internal transcribed spacer 2 (ITS2) gene sequences.

**Findings:**

DNA was extracted from morphologically identified *An. barbirostris* specimens from Sri Lanka, the COI and ITS2 regions amplified and their sequences analysed by comparison with other GenBank entries. Maximum likelihood trees suggested that Sri Lankan *An. barbirostris* constitute a different molecular type most closely related to clade I.

**Conclusions:**

Considering the uncorrected p distances between the clade I and Sri Lankan specimens it is fair to assume that the specimens collected from widely separated locations in Sri Lanka with morphology characteristic of *An. barbirostris s.l*. form a new molecular type with close resemblance to *An. barbirostris s.s* from Indonesia and Thailand.

## Background

*Anopheles barbirostris* van der Wulp is a vector of malaria in Sri Lanka [1], India and Southeast Asia, and a vector of Brugian filariasis in Southeast Asia [2]. *Anopheles barbirostris* and 12 related species form the taxonomically complex Barbirostris Group of malaria vectors in this region ([3] and references therein). *An. barbirostris s.l*. is a species complex with up to five sibling species whose individual distributions are not well established [4–7]. Members of this complex are typically zoophillic, develop in still or slow moving fresh water such as in swamps and rice fields, and are relatively tolerant of organic pollution ([3] and references therein). Larvae were recently also found in brackish water (salinity of 4–15 parts per thousand) in domestic wells and mangrove swamps in the Jaffna district of Northern Sri Lanka [8]. Morphological differentiation of anopheline sibling species is difficult and has previously led to significant misidentification with implications for vector control, e.g. mosquitoes long identified morphologically as *Anopheles subpictus* sibling species B in Sri Lanka were recently shown to be *Anopheles sundaicus s.l*. by analysis of ribosomal RNA gene sequences [9].

Other members of the Barbirostris Group, e.g. *An. reidi* and *An. barbrumbosus*, have been identified in Sri Lanka [10] but the five other species of the Barbirostris Subgroup, *viz. An. campestris, An. donaldi, An. franciscoi, An. hodgkini* and *An. pollicaris* [11] have not been reported in Sri Lanka. The present study was designed to characterize morphologically identified *An. barbirostris s.l.* in Sri Lanka using DNA sequences of the cytochrome oxidase subunit I (COI) of mtDNA and internal transcribed spacer 2 (ITS2) of ribosomal RNA in relation to previous DNA sequence data from *An. barbirostris s.l.* collected elsewhere in Southeast Asia [6].

## Methods

### Mosquito collection

**Figure 1:**
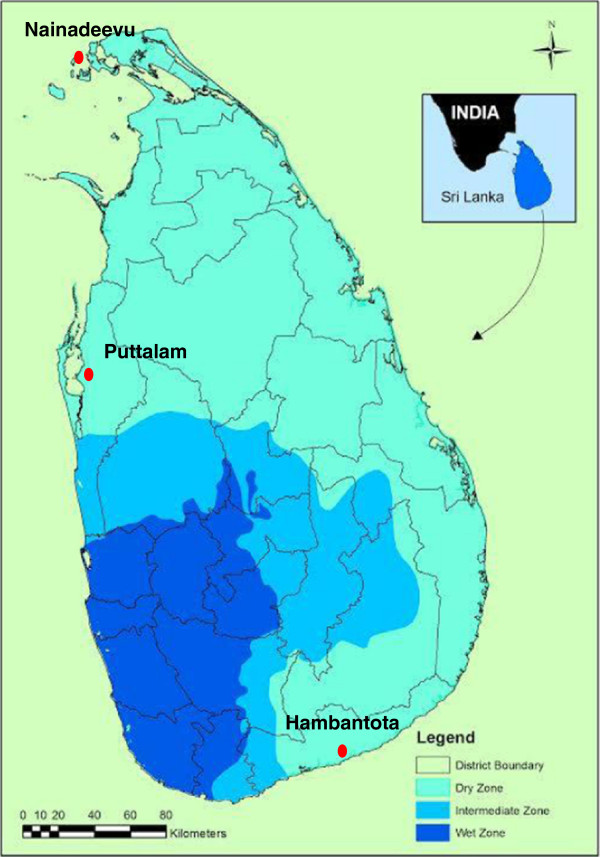
**Map of Sri Lanka showing** ***Anopheles barbirostris*** **collection sites.**

Anopheline mosquitoes were collected from the widely separated locations of Hambantota, Nainadeevu and Puttalam in Sri Lanka (Figure 1) from January to July, 2012 using cattle baited traps. Mosquitoes were identified based on their morphology using taxonomic keys created for Sri Lankan anopheline mosquitoes [12].

### DNA extraction and PCR amplification of COI and ITS2 regions

Among 27 morphologically identified *An. barbirostris s.l*., DNA was extracted from two, one and two specimens collected from Hambantota, Nainadeevu and Puttalam respectively using previously reported methods [13]. The COI region was PCR amplified with forward 5′ TTG ATT TTT TGG TCA TCC AGA AGT 3′ and reverse 5′ TAG AGC TTA AAT TCA TTG CAC TAA TC 3′primers [6]. Each PCR (50 μL total volume) reaction mixture contained 1x Thermo Scientific Ready Mix (1.5 mM Mg Cl_2_, 0.625U Taq DNA polymerase, 0.2 mM dNTPs and 1x Taq buffer), 2-4 μl of template DNA and 10pmoles of each primer. The thermal profile for PCR was 94°C for 5 min, followed by 35 cycles of 95°C for 40 s, 50°C for1 min and72°C for1 min. A final extension temperature of 72°C was set for 10 min.

The ITS2 region was amplified using forward 5′ ATC ACT CGG CTC ATG GAT CG 3′ and reverse 5′ ATG CTT AAA TTT AGG GGG TAG TC 3′ primers [6]. Fifty μl reaction volumes were prepared with 1x reaction buffer, 2.5 mM MgCl_2_; 0.2 mM dNTPs, 20pmoles of each primer, 1.0 U of Taq polymerase and 5 μl of template DNA. The PCR thermal profile was 94°C for 5 min, followed by 35 cycles of 94°C for 1 min, 55°C for 2 min, 72°C for 2 min and a final extension at 72°C for 10 min. The amplified products were separated by agarose gel electrophoresis, stained with ethidium bromide and sequenced at Macrogen, Amsterdam, Netherlands.

Sequences were edited in Finch TV (Geospiza, Seattle, USA) and aligned with Clustal W (MEGA 5.1) [14]. A phylogenetic tree of the sequences was created using the maximum likelihood approach (Phyml 3.0) [15]. A HKY substitution model was selected with a gamma distribution based on the lower Bayesian criterion index (MEGA 5.1) [14]. Genbank deposited sequences from Thailand (16 for COI and 28 for ITS2) and Indonesia (08 samples for COI and 06 samples for ITS2) were also used in the tree construction [5, 6].

## Findings

*Anopheles barbirostris* specimens were identified based on standard morphological characteristics [12]. The presence of a median tuft was observed in all flies. Median scales in the abdominal sternum were also observed. Maxillary palps were dark-scaled and the wings had a narrow apical fringe spot at the vein R_4+5_.

**Figure 2:**
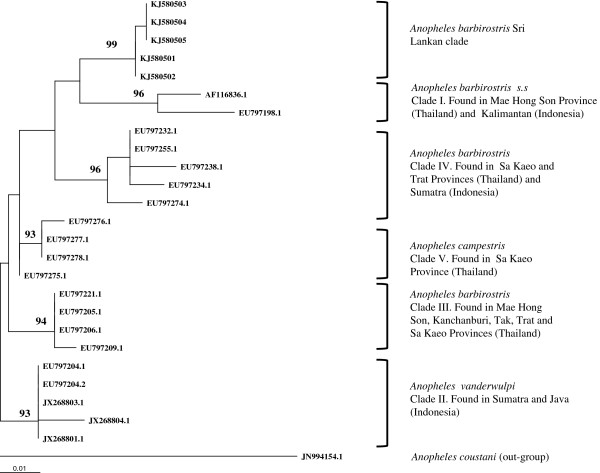
**Maximum Likelihood tree created from CO1 sequence data (400 bp) using Phyml**[15]**with the HKY + G + I substitution model.** The nodes are shown with their bootstrap values; *An. coustani* was used as the out-group. The Genbank accession numbers for Sri Lankan samples are KJ580501 - KJ580505.

All the chromatograms were clean and the ambiguous ends were removed before sequence analysis. The phylogenetic tree (Figure 2) constructed with 400 bp of COI sequence data, including those from previously published sequences, confirmed that all five sequenced Sri Lankan specimens were members of the Barbirostris Complex. Sri Lankan specimens formed a sister clade to clade I of Paredes-Esquivel *et al*. [6], which is *An. barbirostris s.s*. Compared to sequences from the most closely related clade I, the Sri Lankan specimens showed 5.1% variation (with 20 variable sites) in the COI sequences. All the mutations were silent except two where tyrosine was replaced by serine and alanine replaced by proline. Intra-specific variations (0.4%), not associated with specific location, were observed among the Sri Lankan COI sequences.

**Figure 3:**
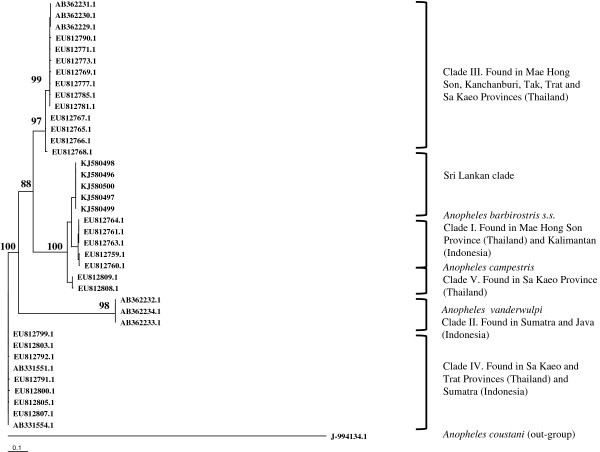
**Maximum likelihood tree created from ITS2 sequence data (580 bp) using Phyml**[15]**with the HKY + G + I substitution model.** The nodes are shown with their bootstrap values; *An. coustani* was used as the out-group. The GenBank accession numbers for Sri Lankan samples are KJ580496 - KJ580500.

The maximum likelihood tree (Figure 3) created with 580 bp of ITS2 sequence data varied slightly from the COI tree but the Sri Lankan specimens remained most closely related to clade I. Compared to sequences from the most closely related clade I, the Sri Lankan specimens showed 5.4% variation (with 28 variable sites including 6 indels). The ITS2 sequences from all five Sri Lankan specimens were identical.

All the nodes in both trees (Figures 2 and 3) were well supported with strong bootstrap values. The maximum likelihood trees created with HKY + G + I model in Phyml were in agreement with the Bayesian trees (data not shown).

*Anopheles barbirostris* were previously classified into five distinct clades by Paredes-Esquivel *et al*. [6], based on COI and ITS2 data. Clade IV was identified as a species with intermediate morphology between *An. barbirostris* and *An. campestris*. Later, clade I was identified unequivocally as *An. barbirostris s.s.* and Clade II was described as a new species named *An. vanderwulpi* [16]. Saeung *et al*. [5] recognized three sibling species in the *An. barbirostris* complex based on karyotyping and COI, COII and ITS2 sequences. The sibling species designated A1 and A2 by Saeung *et al*. [5] correspond to clades III and IV, respectively, of Paredes-Esquivel *et al*. [6]. Differential susceptibility of the members of the Barbirostris Complex to infection with malaria parasites has been established in laboratory reared colonies in Thailand [17].

The disparity between mitochondrial (COI) and nuclear gene (ITS2) trees in the present analysis may be explained in part by different evolutionary rates. Mitochondrial DNA typically mutates at a higher rate than nuclear sequences [18] such as the ITS2, and thus the latter may be more appropriate for analysing relationships between species.

## Conclusions

Considering the uncorrected p distances of 4.5% (COI) and 5.1% (ITS2) between the clade I and Sri Lankan specimens it is fair to assume that the specimens collected from widely separated locations in Sri Lanka with morphology characteristic of *An. barbirostris s.l*. form a new molecular type with close resemblance to the clade I (*An. barbirostris s.s*) of Paredes-Esquivel *et al*. [6].
